# RETRACTION: The Outcome of Prolonged Postoperative Antibiotics on Wound Healing in Orthognathic Surgery: A Meta‐Analysis

**DOI:** 10.1111/iwj.70227

**Published:** 2025-02-11

## Abstract

**RETRACTION:** LuY. and LiJ. , “The Outcome of Prolonged Postoperative Antibiotics on Wound Healing in Orthognathic Surgery: A Meta‐Analysis,” International Wound Journal 20, no. 6 (2023): 2233–2240, 10.1111/iwj.14104.36919189 PMC10333044

The above article, published online on 14 March 2023, in Wiley Online Library (http://onlinelibrary.wiley.com/), has been retracted by agreement between the journal Editor in Chief, Professor Keith Harding; and John Wiley & Sons Ltd. Following an investigation by the publisher, all parties have concluded that this article was accepted solely on the basis of a compromised peer review process. The editors have therefore decided to retract the article. The authors did not respond to our notice regarding the retraction.

**RETRACTION:** Y. Lu and J. Li , “The Outcome of Prolonged Postoperative Antibiotics on Wound Healing in Orthognathic Surgery: A Meta‐Analysis,” International Wound Journal 20, no. 6 (2023): 2233–2240, 10.1111/iwj.14104.36919189 PMC10333044

The above article, published online on 14 March 2023, in Wiley Online Library (http://onlinelibrary.wiley.com/), has been retracted by agreement between the journal Editor in Chief, Professor Keith Harding; and John Wiley & Sons Ltd. Following an investigation by the publisher, all parties have concluded that this article was accepted solely on the basis of a compromised peer review process. The editors have therefore decided to retract the article. The authors did not respond to our notice regarding the retraction.

